# Time Estimation or Autonomic Heart Rate Regulation: Which Mechanism Is More Sensitive in the Development of Internet Addiction in Adolescents?

**DOI:** 10.3390/ijerph191911977

**Published:** 2022-09-22

**Authors:** Olga Krivonogova, Elena Krivonogova, Liliya Poskotinova

**Affiliations:** N. Laverov Federal Center for Integrated Arctic Research of the Ural Branch of the Russian, Academy of Sciences, 163069 Arkhangelsk, Russia

**Keywords:** Internet addiction, time perception, adolescents, heart rate variability

## Abstract

The aim of the study was to assess different combinations of time estimation ability and heart rate variability (HRV) parameters in adolescents during developing of Internet addiction (IA). The study included adolescents aged 16–17 (*n* = 49) living in the southern region of Russia. IA was measured using the Chen Internet Addiction Scale (CIAS). An individual minute test (IM) was performed, and HRV was recorded. There are three groups that differ in HRV, IM duration and CIAS parameters. Minimal and moderate risk of IA development was detected against a background of a tendency towards vagotonia and prolonged IM time (Group I) and balanced autonomic nervous balance and optimal IM time (Group II). A balanced autonomic nervous balance and prolonged IM time were detected in the group of persons with a moderate risk of IA and a stable IA pattern (Group III). We assume that the development of IA in adolescents may be carried out by different neural mechanisms, including optimal autonomic nervous balance, but with primary impairment of cortical brain mechanisms of time perception.

## 1. Introduction

Internet addiction is a common problem today, especially for teenagers. Adolescence, due to hormonal changes, is characterised by increased emotionality, impulsivity and aggressiveness. Lack of self-control at this age can lead to unpredictable and harmful consequences [1]. Ease of access to a variety of information is an advantage of using the Internet, but excessive use can lead to Internet addiction [2]. Internet addiction is defined as the loss of control over behaviour on the internet, leading to psychological, social and professional problems, and maladaptation in everyday life [2]. One of the main characteristics of the problem is the loss of control over the amount of time spent online [2].

The ability to accurately determine the duration of events is important for self-monitoring behaviour [3]. The theory proposed by Craig suggests that the perception of time is a function of integrated interoceptive signals; thus, various physiological factors, such as arousal and body temperature, can affect the perception of time [4,5]. The modulators of time perception are attention, memory and emotional state [6]. The accuracy of time estimation is related to the autonomic nervous system (ANS) [7]. One of the indicators of ANS activity is heart rate variability (HRV) [8]. According to the neurovisceral integration model, as well as the polyvagal Porges theory, HRV is associated with concomitant factors of self-control, such as cognitive and emotional regulation and social activity [9]. It is assumed that internet addiction causes the activation of the sympathetic nervous system [10]. There is evidence that individuals with IA have higher sympathetic activity and lower parasympathetic activity [11,12]. Our previous studies have also shown that increased risk of Internet addiction is accompanied by impaired time estimation in the form of lengthened individual minute time and sympathicotonia in 16–17-year-olds living in the Arctic region of Russia [13]. However, sympathicotonia may not always accompany a high risk of Internet addiction. There is evidence showing that both increased sympathetic activity and activation of the parasympathetic nervous system are observed in individuals at high risk of developing IA [10]. It may be due to high functional reserves of vagal regulation that compensates a stress-related sympathicotonia, especially in adolescents. The hypothesis is that autonomic nervous mechanisms of cardiac activity may remain optimal in the early stage of Internet addiction risk development, but that time perception will be a more sensitive mechanism to excessive use of Internet resources. This is because the mechanisms of cortical brain regulation may be primarily impaired, rather than deep brain structures involved in the regulation of visceral organs, including the heart. Thus, the aim of the study was to assess different combinations of time estimation ability and heart rate variability (HRV) parameters in adolescents during the development of Internet addiction.

## 2. Materials and Methods

In April 2021, the study involved healthy young people aged 16–17 (*n* = 49; 32 girls, 17 boys) who were students at the school of the Southern Federal District of the Russian Federation.

The participants were a group of healthy individuals according to medical criteria based on the conclusions of a paediatrician. All participants successfully completed the educational school programme and coped with the school curriculum. Insomnia as a medical diagnosis was not verified in the study participants. The study participants had not committed any offences and did not need police or psychological supervision in connection with the manifestation of aggression, depression, suicidal behaviour or the use of psychoactive substances. Body mass index (BMI) was defined as the body mass divided by the square of the height (kg/m^2^), with mass in kilograms and height in metres. Height, weight and BMI were measured using certified medical equipment with electronic scales and a height meter (REP+VMEN-200-100-D1-A, Twes Company, Tambov, Russian Federation). The anthropometric parameters of the examined persons were above the 97th and below the 3rd percentiles in accordance with the growth-age scales for 5–19 years and BMI age for 5–19 years for the examined persons of the appropriate age, in accordance with the criteria of the World Health Organization [14].

Internet addiction behaviour was assessed with the Chen Internet Addiction Scale (CIAS) [15] using the Russian version that was developed by Malygin and Feliksov [16]. When studying Internet addiction, the reliability measures of the CIAS test (Cronbach’s alpha) ranged from 0.757 to 0.9, depending on the CIAS subscales [16]. In the present study, Cronbach’s alpha test values ranged from 0.75 to 0.78 depending on the CIAS scales, indicating sufficient reliability of these data.

Symptoms of IA include compulsive symptoms (Com), withdrawal (Wit), tolerance (Tol), interpersonal and health-related problems (IH-RP), and time management (Tm) problems. The CIAS comprises a questionnaire with 26 items and a four-point Likert scale, ranging from 1 point (Does not match my experience) to 4 points (Definitely matches my experience). Thus, the minimum CIAS value is 26, and the maximum value is 104. Respondents with CIAS scores above 64 points were considered to have a stable pattern of Internet addiction, scores of 43 to 64 were associated with a moderate risk of developing IA, and those with less than 43 points indicated a minimal risk of developing IA. The preliminary analysis did not reveal statistically significant differences between the CIAS scores in males and females; therefore, all of the indicators are presented for the general sample.

The individual minute test was used to estimate the time [17]. When assessing the duration of an individual minute (IM), the researcher indicated the start to the participant, who then counted down the seconds from 1 to 60, while the researcher monitored the seconds of physical (real) time on the stopwatch. When the participant reached 60 s of individual time, they indicated the end of the count aloud, and the researcher stopped the stopwatch. The time on the researcher’s stopwatch was regarded as the participant’s time.

HRV was assessed using the “Varicard” equipment (Varicard Company, Ryazan, Russian Federation). Mood and emotional reactions can affect the autonomic nervous activity of study participants. Therefore, before starting the study, we recommended the participants to spend 5–10 min in a state of relaxation and rest. We calculated HRV indices after removing artifact and extra systolic cardiac intervals. HRV indices were recorded with the participant in a sitting position for 5 min. To characterise the heart rate (HR), we used the average rate for 5 min, recorded using a cardiointervalogram. The HRV indicators used included HR (bpm), standard deviation of N-N intervals (intervals between normal R peaks) (SDNN, ms), the root-mean-squared differences in successive N-N intervals (RMSSD, ms), and pNN50 % is the proportion of NN50 count (number of pairs of adjacent NN intervals differing by more than 50 ms) divided by the total number of all NN intervals. SDNN indicates total HRV, while RMSSD and pNN50% are used to predict parasympathetic activity [18]. Data of variation pulsimetry are used to calculate the widespread in Russia index of regulation straining or stressindex (SI). SI was calculated by the formula SI = AMo/2Mo × MxDMn:Mo (mode), AMo (mode amplitude), and MxDMn (variation range). Index of regulation strain (SI) characterizes the activity of sympathetic or central regulation [19]. Frequency-domain measurements estimate the distribution of absolute power into three frequency bands: high-frequency range of HRV spectrum (0.15–0.4 Hz, HF, ms2) representing largely the parasympathetic influence on heart rate; low frequency range of HRV spectrum (0.04–0.15 Hz, LF, ms2 reflects parasympathetic and sympathetic nerve activity and its effects on heart rate; very low frequency range of HRV spectrum (0.04–0.003 Hz, VLF, ms2) conditioned, first and foremost, by the humoral influence on heart rate. Total power (TP, ms2) is a sum of power in the HF, LF and VLF ranges [19]. 

Statistical processing of the materials was carried out using the Statistica 10.0 program (StatSoft, Tulsa, OK, USA). Due to the fact that the vast majority of the studied parameters were not normally distributed, they are described using of the median and range of values corresponding to the 25th and 75th percentiles (lower and upper quartiles). Cluster analysis was performed using the k-means method and z-transformation of the data was performed in order to reduce the asymmetry in the distribution of the variables. Comparison of quantitative variables between several independent groups was carried out using the Kruskal–Wallis criterion. Then, in order to clarify which groups showed differences, a pairwise comparison using the Mann–Whitney Utest was used, with the correction of the significance criterion for multiple comparisons. For three groups, the critical significance level was *p* < 0.017.

## 3. Results

Using cluster analysis, three homogeneous groups were identified, differing in HRV parameters, IM duration and CIAS scores (Table 1). The medians and interquartile range for CIAS values in Groups I and II were similar, but the maximum CIAS values in Groups I and II were up to 50 score. This indicates that the groups I and II included people with both minimal and moderate risk of Internet addiction (43 points or higher).

The values of the CIAS and the Com, Wit, Tol, Tm and IH-RP scales were higher in Group III compared to groups I and II. The interquartile range of the CIAS value reflects the presence in Group III of individuals with both moderate risk of Internet addiction and individuals with a stable IA pattern (64 points or higher). Against the background of similar quantitative CIAS and CIAS subscale values, the time assessment by study participants in groups I and II performed differently. Thus, the IM duration was significantly longer in Group I than in Group II. In Group III, the IM was longer than in Group II (60–72 s in 25th–75th percentiles). The groups differed in the temporal and spectral characteristics of HRV (Table 2).

Group I individuals have the highest total HRV spectrum power (TP) and the highest vagus regulation indices as rMSSD, pNN50, SDNN and HF. Group II subjects tended to be sympathicotonia in SI, and they had the least vagal activity (as measured by HF, pNN50 and rMSSD). In Group I, the upper quartile of SI value was below 80 units, which indicates the predominance of the activity of the parasympathetic nervous system in the regulation of heart rhythm [19]. In persons of groups II and III, the interquartile ranges of SI values were (88.8; 224.2) and (86.2; 161.8) units, respectively, which reflects a relative shift towards a sympathetic tone. However, in Group III, such a shift occurs against the background of maintaining vagal parameters at a level similar to that in Group I (according to TP, rMSSD, SDNN values). 

## 4. Discussion

According to our research, the formation of Internet-addicted behaviour can occur against the background of a balanced autonomic tone without pronounced sympathicotonia. In persons of Group II, the minimal and moderate risk of developing IA was accompanied by the time of an individual minute close to the optimal for this age (50–59 s). This may indicate a relative adaptation of these adolescents to the risks associated with excessive use of the Internet, and the absence of obvious violations of both autonomic neural mechanisms and brain mechanisms of time estimation. However, there may be a violation of the perception of time, which is expressed in the form of an underestimation of time against the backdrop of a tendency to vagotonia (an increase in the time of IM in Group I persons). A more pronounced risk of developing IA and even the presence of a stable IA pattern may also be accompanied by the preservation of vagal reserves of autonomic regulation of the heart rate and the absence of pronounced sympathicotonia, but there will also be a violation of time estimation (an increase in the time of IM in Group III persons). Such data indicate a higher sensitivity of the brain mechanisms of time perception than the mechanisms of autonomous nervous regulation of the visceral organs to early signs of the formation of Internet addiction. 

Various brain regions and neural networks have been proposed to participate in the perception of time [6]. The basal ganglia, supplementary motor area, cerebellum, prefrontal cortex, inferior parietal cortex and insular cortex, as well as the inferior parietal and insular cortex were proposed as responsible for the perception of time [20]. A number of studies have shown that the basal ganglia and supplementary motor area are associated with timekeeping mechanisms, while the frontal–parietal network may be associated with the processes of attention and memory necessary for the perception of time [21].

According to the results of our study, the activity of the parasympathetic nervous system in the regulation of heart rhythm and an elongated individual minute (underestimation of time) prevailed in Group I, including persons with moderate IA risk. The Craig theory suggests that the perception of time is formed in the insula cortex, which is involved in both visceral integration and autonomic control [5,6]. It is assumed that an increase in the activity of the parasympathetic nervous system is accompanied by increased activation of the anterior part of the left side of the insula cortex. This combination of activation leads to a reduction in time and, consequently, to a subjectively shortened perception of duration, i.e., underestimation of time [3]. In our study, no autonomic nervous imbalance was detected in Group III. However, the lengthening of the individual minute was noted. A number of studies have reported an increase in sympathetic activity and a decrease in parasympathetic activity in Internet addiction disorders [11]. In another study, no sympathovagal imbalance was found in people with Internet addiction disorders [22], as in our study; the authors assumed that the study participants did not have severe Internet addiction disorders. In our previous studies, when using a similar methodology in adolescents aged 16–17, we also observed significant sympathicotonia and underestimation of time with a similar CIAS score [13]. Apparently, the degree of autonomic nervous disorders also depends on the environmental conditions where Internet users live.

The literature shows that time distortion has been noted in association with addictive behaviour, with results showing that dependence is associated with stronger temporary misperception of time, with users underestimating time intervals [23]. In participants with internet addiction, neuroimaging studies revealed changes in the frontal areas of the brain (orbitofrontal and prefrontal cortex) and the brain reward system (putamen and nucleus accumbens) [24]. It is assumed that the centre of integration of time and reward networks may be the prefrontal–striatal circuit, with the participation of dopaminergic and glutamatergic neurons [20].

## 5. Conclusions

The formation of signs of Internet addiction (both the minimal, moderate risk of development and the presence of a stable pattern of IA) in adolescents may be against the background of various variants of the autonomic balance, including a tendency to vagotonia and a balanced autonomic tone, but with a primary disturbance in the time estimation. This is due to the activation of various neuronal pathways, both the rhythm-setting structures of the cerebral cortex and the centres of autonomic nervous regulation of visceral organs, which support and ensure addictive behaviour in adolescents. The significance of the performed study lies in the need to identify early signs of Internet addiction in adolescents, not only according to the criteria for impaired autonomic regulation of the heart rhythm, but primarily in determining the time estimation. Underestimation of subjective time may be an early marker of Internet addiction, including against the background of vagotonia.

The limitations of the study included the small study sample, the lack of detailed analysis of online activity, and the use of simple statistical methods, due to a wide range of individual reactions of the persons at early signs of the risk of Internet addiction.

## Figures and Tables

**Table 1:** CIAS subscale scores and IM duration in adolescents with different risks of IA ^a^.

Variables	Group I, *n* = 14	Group II, *n* = 17	Group III, *n*= 18	H Test, Kruskal–Wallis	*p* Kruskal–Wallis
	1	2	3		
CIAS, score	40 (33; 45)	39 (33; 43)	59 (52; 67) ^b (1–2, 1–3)^	29.6	0.001
Com, score	8 (6; 9)	6 (6; 9)	12.5 (11; 14) ^b (1–3, 2–3)^	28.3	0.001
Wit, score	8 (6; 9)	8 (7; 10)	13.5 (12; 15) ^b (1–3, 2–3)^	27.7	0.001
Tol, score	7 (6; 9)	6 (5; 8)	10.5 (9; 12) ^b (1–3, 2–3)^	16.8	0.001
IH-RP, score	8.5 (7; 11)	9 (8; 11)	14 (10; 16) ^b (1–3, 2–3)^	17.8	0.001
Tm, score	7 (6; 8)	6 (5; 9)	10 (8; 13) ^b (1–3, 2–3)^	15.5	0.001
IM, sec	73 (66; 84)	55 (50; 59) ^b (1–2)^	64.5 (60; 72) ^b (2–3)^	23.2	0.001

CIAS: Chen Internet Addiction Scale; IM: individual minute; IA: internet addiction; Com: compulsive use; Wit: withdrawal symptoms, Tol: tolerance; IH-RP: interpersonal and health-related problems; Tm: time management problems. ^a^ Data are presented as median (lower and upper quartiles). ^b^ Statistically significant difference between the groups: *p* < 0.001, the Mann–Whitney *U* test.

**Table 2:** Heart rate variability parameters in 16–17-year-old adolescents with different risks of IA ^a^.

Variables	Group I, *n* = 14	Group II, *n* = 17	Group III, *n* = 18	H Test, Kruskal–Wallis	*p* Kruskal–Wallis
	1	2	3		
HR, bpm	78.1 (67.3; 84.3)	82.6 (76.5; 87.5)	84.7 (79.1; 88.3)	3.14	0.181
rMSSD, Ms	51.5 (44.2; 66.2)	32.9 (20.2; 34.8) ^c (1–2)^	34.3 (30.1; 46.4)	11.1	0.002
pNN50, %	33.9 (24.2; 37.8)	10.1 (2.1; 14.1) ^c (1–2)^	13.2 (9.6; 27.3)	11.5	0.002
SDNN, Ms	64.3 (56.7; 81.1)	43.1 (36.1; 55.1) ^c (1–2)^	52.7 (42.2; 62.4)	9.7	0.004
SI, units	64.1 (35.5; 77.1)	127.6 (88.8; 224.2) ^b (1–2)^	104.5 (86.2; 161.8)	7.9	0.011
TP, ×1000, Ms^2^	3.5 (2.9; 6.9)	1.7 (1.2; 2.4) ^c (1–2)^	2.1 (1.4; 3.2) ^b (1–3)^	12.7	0.001
HF, ×1000, Ms^2^	1.7 (1.2; 2.2)	0.4 (0.2; 0.6) ^c (1–2)^	0.7 (0.6; 1.1) ^c (2–3)^	14.8	0.001
LF, ×1000, Ms^2^	1.1 (0.8; 2.8)	0.5 (0.4; 0.9) ^b (1–2)^	0.7 (0.5; 1.1) ^b (1–3)^	10.6	0.005
VLF, ×1000, Ms^2^	0.4 (0.2; 0.8)	0.2 (0.1; 0.5)	0.3 (0.2; 0.6)	2.1	0.255

HR: heart rate; SDNN: standard deviation of all NN intervals; rRMSSD: the square root of the mean squared differences in successive NN intervals; pNN50: percentage of the number of pairs of consecutive cardio intervals differing by more than 50 ms; SI: stress index; TP: total power of HRV spectrum; HF: high frequency range of HRV spectrum; LF: low frequency range of HRV spectrum; VLF: very low frequency range of HRV spectrum. ^a^ Data are presented as median (lower and upper quartiles). ^b^ Statistically significant difference between the groups: *p* < 0.01, the Mann–Whitney Utest. ^c^ Statistically significant difference between the groups: *p* < 0.001, the Mann–Whitney *U* test.

## Data Availability

Not applicable.
